# Investigation into the potential mechanism and molecular targets of Fufang Xueshuantong capsule for the treatment of ischemic stroke based on network pharmacology and molecular docking

**DOI:** 10.3389/fphar.2022.949644

**Published:** 2022-09-15

**Authors:** Lei Wang, Liping Wang, Hui Wang, Ting Zhu

**Affiliations:** ^1^ Institute of Neuroregeneration and Neurorehabilitation, Department of Pathophysiology, School of Basic Medicine, Qingdao University, Qingdao, China; ^2^ School of traditional Chinese pharmacy, China Pharmaceutical University, Nanjing, China; ^3^ Pharmacy Intravenous Admixture Services, Qingdao Women and Children's Hospital, Qingdao, China; ^4^ Changzhi Maternal and Child Health Care Hospital, Changzhi, China

**Keywords:** Fufang Xueshuantong (FFXST) capsule, ischemic stroke (IS), network pharmacology, molecular docking, potential mechanism, molecular targets

## Abstract

Fufang Xueshuantong (FFXST) capsule is a traditional Chinese medicine (TCM) preparation used to activate blood circulation, resolve stasis, benefit qi, and nourish yin in clinical practice. However, its potential mechanism and molecular targets after ischemic stroke (IS) have not been investigated. The aim of this research was to investigate the molecular mechanisms of FFXST in the treatment of IS based on network pharmacology and molecular docking. We used the Traditional Chinese Medicine Systems Pharmacology Database and Analysis Platform (TCMSP) to collect candidate compounds of four herbs in FFXST; disease-related differential genes were screened using the Gene Expression Omnibus (GEO) database, and a compound–disease network was created using Cytoscape 3.8.2 software. The topological analysis of the protein–protein interaction (PPI) network was then created to determine the candidate targets of FFXST against IS. Gene Ontology (GO) and Kyoto Encyclopedia of Genes and Genomes (KEGG) enrichment analyses were conducted using the clusterProfiler package in *R*. The gene–pathway network of FFXST against IS was created to obtain the key target genes. Molecular docking was used to validate the core targets using AutoDock Vina 1.1.2. A total of 455 candidate compounds of FFXST and 18,544 disease-related differential genes were screened. Among them, FFXST targets for IS treatment had 67 active compounds and 10 targets in the PPI network related to STAT1, STAT3, and HIF1A. The biological processes of GO analysis included the regulation of reactive oxygen species metabolic process, cellular response to chemical stress, regulation of angiogenesis, regulation of vasculature development, positive regulation of cytokine production, and response to oxidative stress. The KEGG enrichment analysis showed that Kaposi sarcoma-associated herpesvirus infection, microRNAs in the cancer signaling pathway, Th17 cell differentiation, and HIF-1 signaling pathway were significantly enriched. The network pharmacology outcomes were further verified by molecular docking. We demonstrated that FFXST protection against IS may relate to the regulation of oxidative stress, immune inflammatory response, and angiogenesis through the relevant signaling pathways. Our study systematically illustrated the application of network pharmacology and molecular docking in evaluating characteristics of multi-component, multi-target, and multi-pathway of FFXST for IS.

## Introduction

As estimated by the latest WTO report, stroke is the second leading cause of death in the world, and approximately 87% of stroke-related deaths belong to ischemic stroke (IS) (Zhou et al., 2018). Currently, the only FDA-approved drug for use in ischemic stroke is recombinant tissue plasminogen activator (rt-PA). However, due to the narrow time window for t-PA treatment and the risk of complications, only 3%–5% of patients benefit from t-PA therapy (Dibajnia and Morshead, 2013; Dirnagl and Endres, 2014). Endovascular thrombectomy is a means to improve vascular recanality clinically and is more effective than t-PA (Papanagiotou and Ntaios, 2018). As it has a high risk of surgery and a high postoperative recurrence rate, only a minority of patients can receive surgical intervention. Therefore, the development of drugs for stroke treatment is in urgent need.

Scholars at home and abroad have carried out many studies on the pathogenesis of ischemic stroke and how to prevent and treat the disease. However, the pathogenesis of IS is complicated and is influenced by multiple factors, many of which are still incompletely understood. The mechanisms underlying ischemic stroke are mainly related to excitatory neurotransmitters, Ca^2+^ overload, oxidative stress, energy metabolism disorders, and apoptosis (Zhou et al., 2018; Campbell et al., 2019). In response to the aforementioned aspects, researchers have developed neuroprotective drugs, but there are not many drugs that can achieve the expected therapeutic effect. The main reason lies in the key target proteins, and strategies for the treatment of stroke are too single, and it is difficult to achieve the desired therapeutic effect by intervening and treating the single mechanism and approach involved previously (Adams et al., 2007). This suggested to us that the use of natural medicinal resources to discover drugs that block IS pathways through multichannel and multi-effect and intervene key targets can provide new ideas and approaches for the treatment of IS.

Due to the complexity of the pathogenesis of ischemic stroke, more and more researchers have taken traditional Chinese medicine (TCM) as an important way to treat ischemic stroke (Zhu et al., 2021a; Zhu et al., 2022; Zhu and Wan, 2022). Fufang Xueshuantong (FFXST) capsule is a TCM preparation based on Sanqi jointly developed by Zhongshan Medical College of Sun Yat-sen University and Guangdong Zhongsheng Pharmaceutical Factory, which has the pharmacological effect of activating blood circulation, resolving stasis, benefiting qi, and nourishing yin. The formula of FFXST consists of Sanqi, Danshen, Huangqi, and Xuanshen, and the amount of Sanqi accounts for about 54.3% of the whole formula. Among them, saponin compounds are the main chemical components of *Panax notoginseng*, which have been widely used in the treatment of cerebrovascular diseases, and have the effect of improving blood circulation (Zhu et al., 2021b) in the brain and protecting brain cells (Zhu et al., 2020; Wang L. et al., 2021). Huangqi is a traditional Chinese Qi-tonifying herb. Astragaloside is one of the active ingredients of Huangqi, which has the effects of improving the permeability of the blood–brain barrier, reducing the free radical content of ischemic brain tissue, and inhibiting inflammatory mediators (Kang et al., 2021). Danshen has the effect of removing blood stasis, relieving pain, clearing the heart, and dispelling troubles. At present, numerous studies have shown that Danshen has a cerebral protective effect on ischemic stroke (Wu et al., 2007). Xuanshen is the dry root of *Scrophularia ningpoensis Hemsl.*, which possessed the effects of nourishing Yin and relieving fire.

**FIGURE 1:**
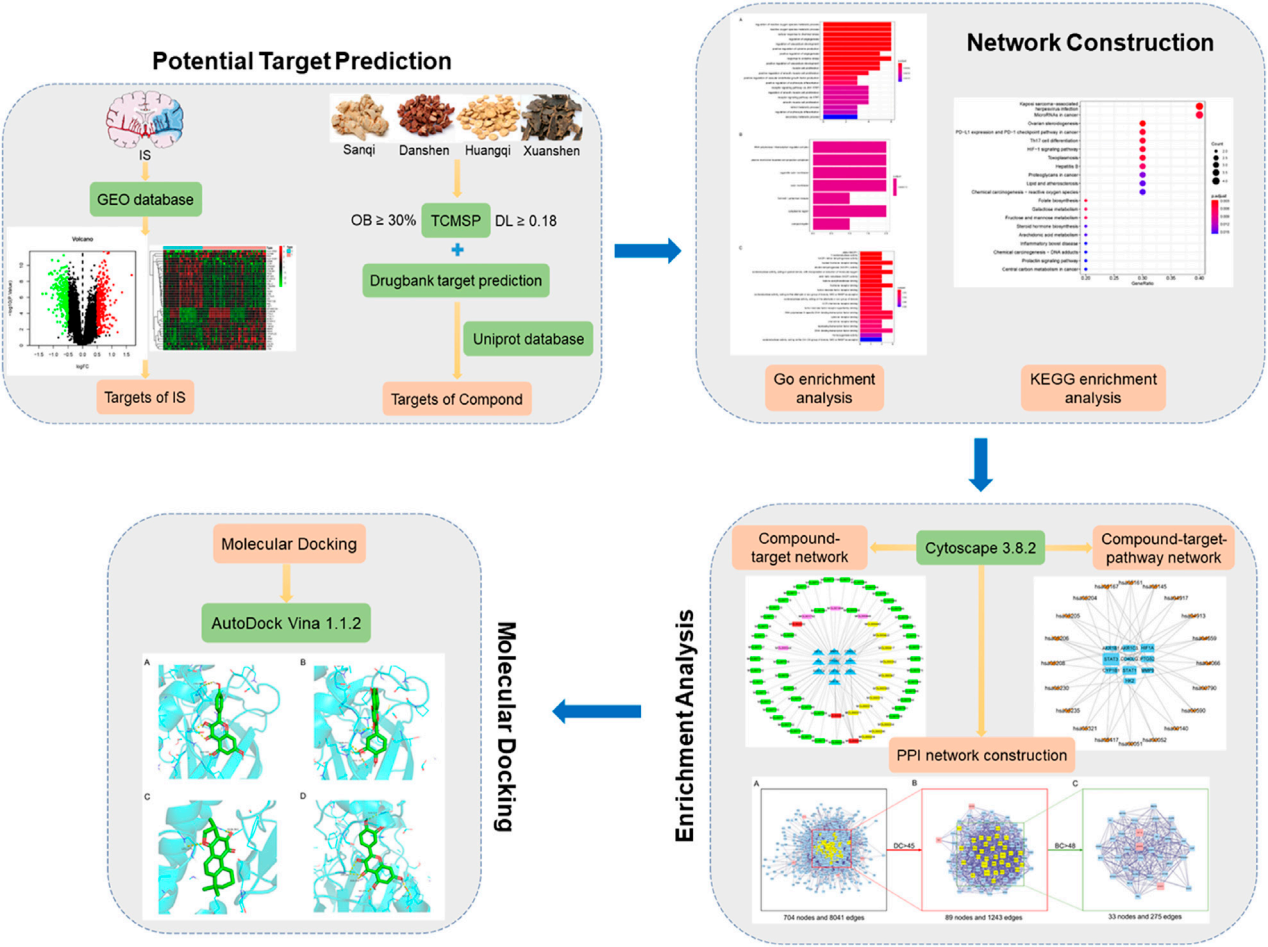
Specific process of network pharmacology analysis.

Owing to its multi-component, multi-target, and multi-pathway synergistic characteristics, TCM compounds can treat various diseases through potential ingredient–target interactions. Network pharmacology is a bioinformatics network construction and network topology analysis strategy, which observes the complex network relationship of “drug–target–disease” from the overall level and then guides the basic and clinical research of TCM, which is systematic and consistent with the overall view of TCM (Zeng et al., 2017; Jiang et al., 2019). In the past few years, the application of network pharmacology in the study of TCM has promoted the transformation of TCM from “single target and single component” to “multi-target and multi-component”, providing a new means for the basic research of pharmacodynamics substances of TCM and the elaboration of the overall mechanism of the therapeutic effect of TCM (Li et al., 2020; Wang K. et al., 2021). In the present study, we explore and analyze the molecular mechanism of FFXST in the treatment of IS based on the method of network pharmacology and molecular docking, in order to provide theoretical reference for subsequent experimental research. The specific process analysis is shown in Figure 1.

## Materials and methods

### Fufang Xueshuantong capsule active ingredient screening and potential target prediction

Through the Traditional Chinese Medicine Systems Pharmacology Database and Analysis Platform (TCMSP, http://lsp.nwu.edu.cn/tcmsp.php), with oral bioavailability (OB) ≥ 30% and drug-like properties (DL) ≥ 0.18 as the filter conditions, the names of FFXST single-flavor Chinese medicines (Sanqi, Huangqi, Danshen, and Xuanshen) were used as keywords to search (Li et al., 2015). The effective chemical components of each single-flavor Chinese medicine in FFXST and their corresponding potential targets were screened out, and the UniProt database (https://www.uniprot.org/) was used to convert the obtained target names into standard gene names to obtain the gene targets of FFXST.

### Acquisition of differential genes

The differentially expressed genes in IS patients were derived from the GEO database (https://www.ncbi.nlm.nih.gov/geo/, Series: GSE16561). The statistical significance of differential expression was set at *p*-value filter < 0.05 and logFC filter > 0.5. The differentially expressed genes presented in the form of a volcano map were depicted using the *R* package, and the top 20 differential genes were selected to render the heat map.

### Compound–disease network construction

Using customized *Perl* scripts, the cross-genes of TCM target genes and disease difference genes were obtained. Combining the active ingredients of TCM, the TCM compound–disease regulatory network was constructed and visualized using Cytoscape 3.8.2 software.

### Protein–protein interaction network construction

The PPI network was constructed and visualized using Cytoscape 3.8.2 software with the *BisoGenet* plugin. The parameter selected was “*Homo sapiens*.” The PPI data were derived from the Interacting Proteins (DIP™), Biological General Repository for Interaction Datasets (BioGRID), Human Protein Reference Database (HPRD), IntAct Molecular Interaction Database (IntAct), Molecular INTeraction (MINT) database, and Biomolecular Interaction Network Database (BIND) (Zhong and Fang, 2020). Visualization of the PPI networks of FFXST-related targets and IS-related targets was carried out using Cytoscape 3.8.2 software.

### Protein–protein interaction network core target screening

The targets with the greatest correlation (core targets) are obtained using Cytoscape CytoNCA plugin according to betweenness centrality (BC), closeness centrality (CC), degree centrality (DC), eigenvector centrality (EC), local average connectivity (LAC), and network centrality (NC) (Jiang et al., 2019).

### Gene ontology and kyoto encyclopedia of genes and genomes pathway enrichment analyses

Using the clusterProfiler package in *R*, the GO and KEGG enrichment analyses were carried out. Enrichment analysis of biological process (BP), cellular component (CC), and molecular function (MF) of potential targets was performed separately. First, the Bioconductor in the R package “org. Hs. eg.db” was installed and run, converting key target genes from each component for the treatment of ischemic stroke into Entrez IDs. Then, the clusterProfiler in the *R* package was installed, the species as a human source was set according to the converted Entrez ID, *p*(*p*-value) < 0.05 was selected for the threshold of the key target gene GO and KEGG analyses, and the top 10 enrichment results were output in the form of histograms and bubble charts.

### Molecular docking

The top three core targets in the PPI network were screened for molecular docking with their corresponding chemical compositions. The PubChem database (https://pubchem.ncbi.nlm.nih.gov/) was used to download small molecule ligand files of chemical composition, and they were imported into Chem3D software for spatial structure conversion and energy optimization. The gene ID of the core target was retrieved from the UniProt database, and the corresponding PDB format file was downloaded from the PDB database (http://www1.rcsb.org/). After water molecule removal and ligand isolation using PyMOL software, the macromolecular receptor file of the obtained core target was imported into AutoDockTools 1.5.7 software for hydrotreating. The docking core target and its corresponding chemical composition were performed using AutoDock Vina 1.1.2. The binding energy was revealed as an evaluation index for molecular docking.

## Results

### Fufang Xueshuantong capsule active ingredient screening and potential target prediction

A total of 119 active ingredients in Sanqi, 87 active ingredients in Huangqi, 202 active ingredients in Danshen, and 47 active ingredients in Xuanshen were screened by the TCMSP database. Ultimately, 102 candidate compounds of FFXST were finally collected with OB ≥ 30% and DL ≥ 0.18 as the screening requirements from the TCMSP, including 8 in Sanqi, 20 in Huangqi, 65 in Danshen, and 9 in Xuanshen (Supplementary Table S1). DrugBank and UniProt databases were used for the prediction of potential targets. Eventually, 716 targets in Sanqi, 953 in Huangqi, 2,565 in Danshen, and 499 in Xuanshen were collected.

### Acquisition of differential genes

**FIGURE 2:**
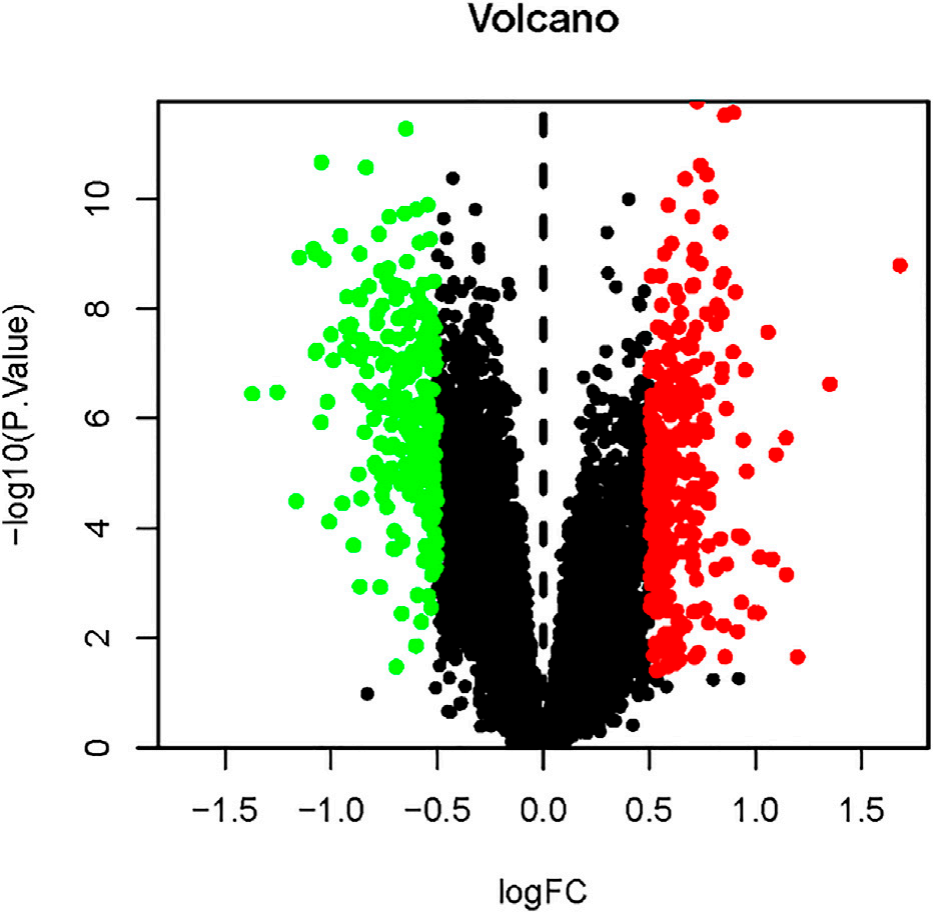
Volcano map of differential gene expression. Upregulated genes are indicated in red, downregulated genes are indicated in green, and black represents genes with no significant changes.

**FIGURE 3:**
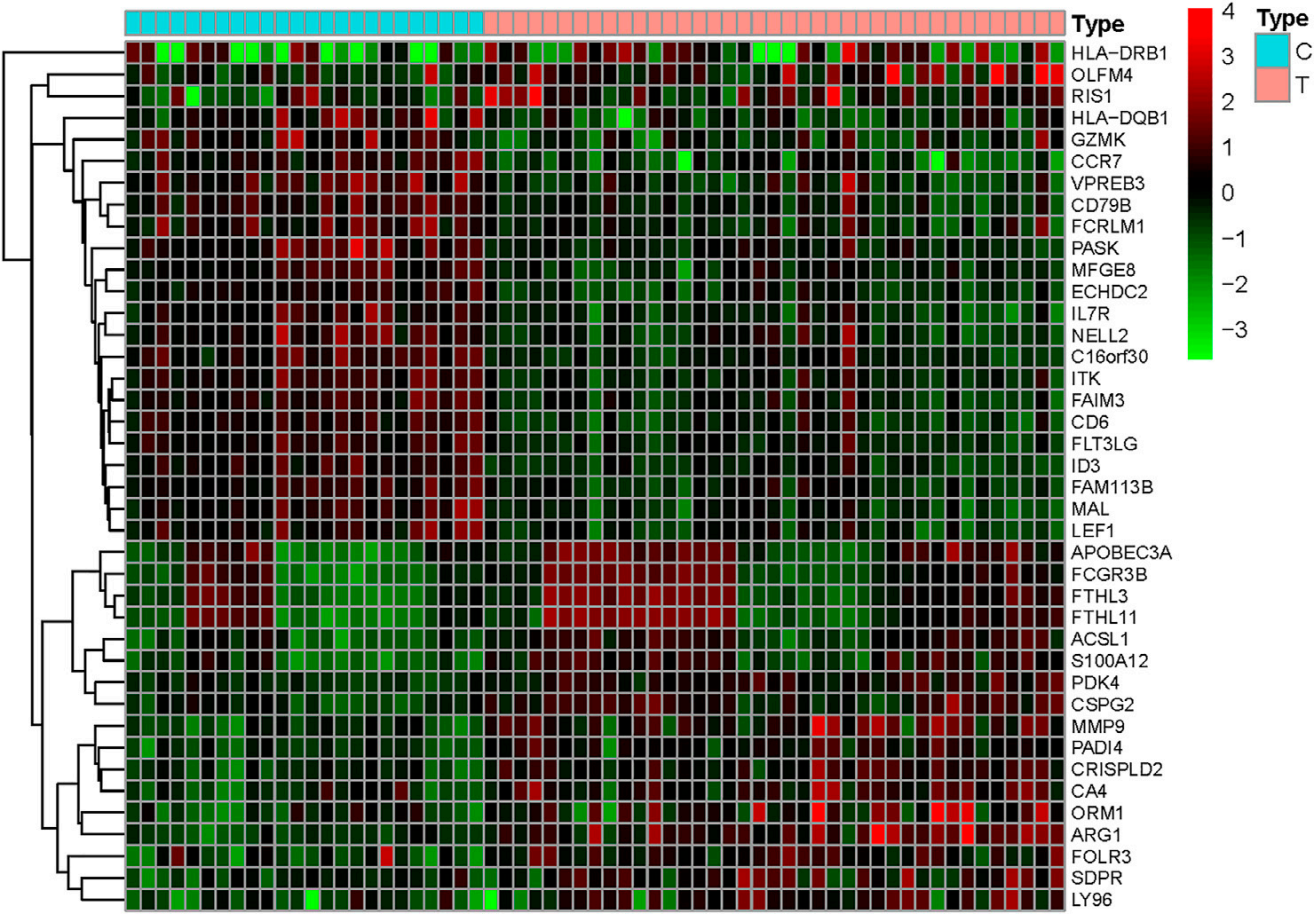
Gene heat map. Upregulated genes are indicated in red (logFC > 0) in the genome, downregulated genes are indicated in green (logFC < 0) in the genome, and black represents genes that do not have significant differences. The first 24 samples came from the control group, and the last 39 samples came from the stroke group.

Comparing 24 normal samples and 39 disease samples in the GEO database, 18,544 disease-related differential genes were screened, including 10,073 upregulated genes and 8,471 downregulated genes. With a *p*-value filter < 0.05 and logFC filter > 0.5 as the screening condition, 282 upregulated genes and 245 downregulated genes were obtained. As shown in Figure 2, the differential genes in the disease samples are normally distributed, and the number of upregulated differential genes is more than that of the downregulated differential genes. The top 20 genes with the most significant up- and downregulation are shown in Figure 3 and Supplementary Table S2.

### Compound–disease network construction

**FIGURE 4:**
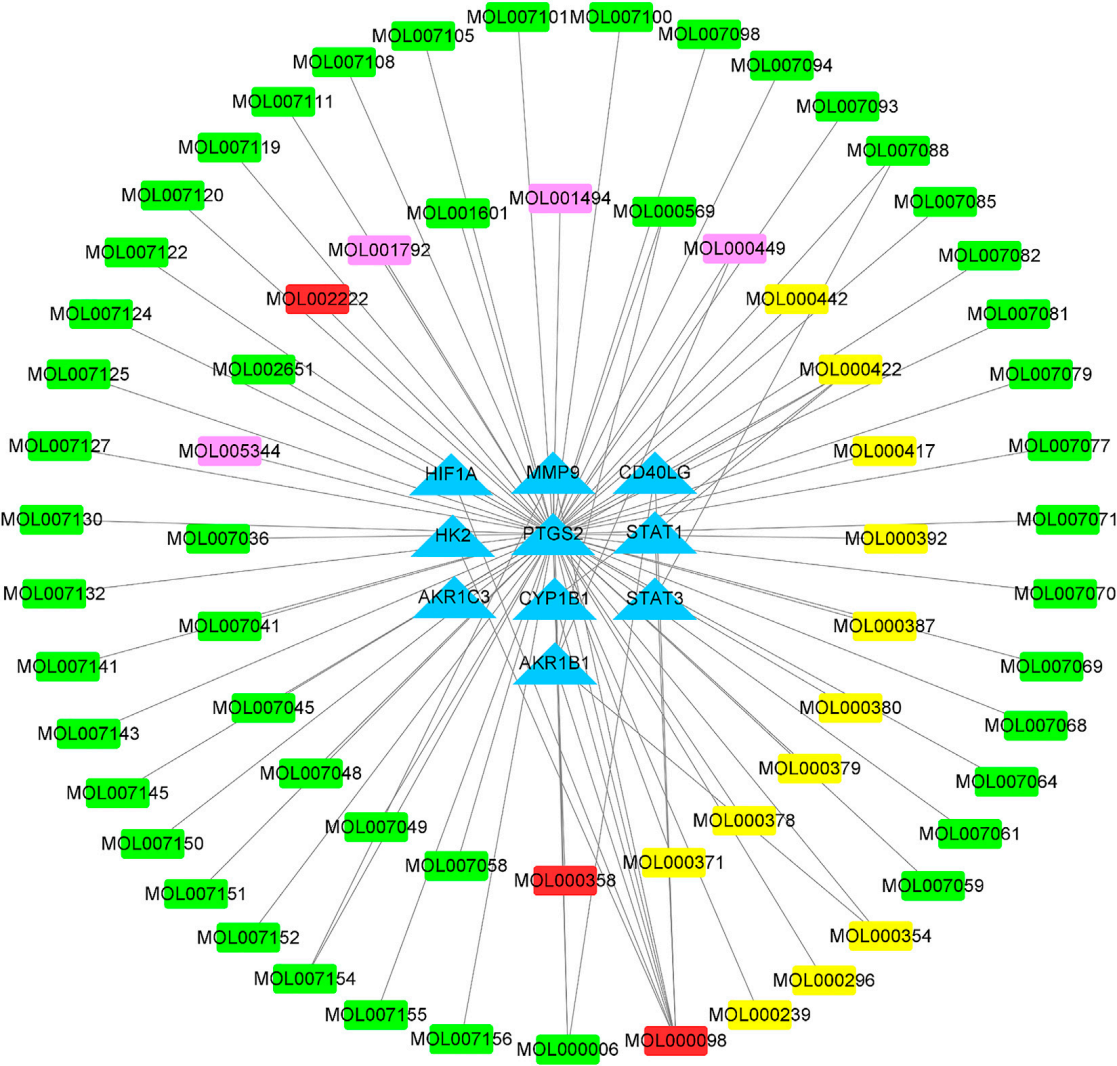
Compound–disease network of FFXST. Green, yellow, and pink squares represent the compounds from Danshen, Huangqi, and Sanqi, respectively; red square represents the multidrug; and blue triangles represent targets.

There are 10 intersection genes as shown in Supplementary Table S3. The FFXST-related target network was created using the collected candidate compounds and compound targets (Figure 4). The network contained 77 nodes (67 compounds and their 10 targets in FFXST) and 84 edges which indicated the FFXST–target interactions. The number of related target genes in the active ingredients of Danshen and Huangqi was the largest, indicating that Danshen and Huangqi in FFXST are the most effective components. Quercetin, kaempferol, and luteolin acted on 8, 5, and 3 targets, respectively. Also, the OB of quercetin, kaempferol, and luteolin is 46.43, 41.88, and 36.16%, respectively. Therefore, they might be the key bioactive compounds of FFXST due to their importance in network position. The *PTGS2* is the gene associated with the highest number of bioactive compounds.

### Protein–protein interaction network core target screening

**FIGURE 5:**
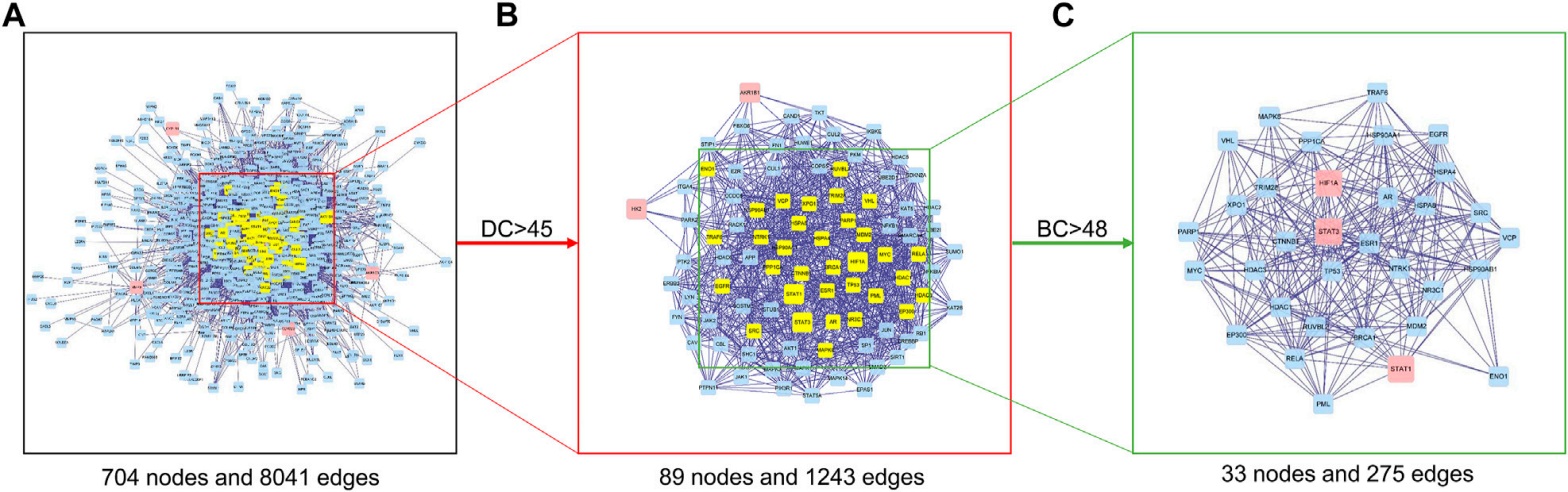
Topological analysis of the PPI network. **(A)** Interactive PPI network of FFXST-related targets and IS-related targets. **(B)** PPI network of significant proteins extracted from **(A)**. **(C)** PPI network of candidate FFXST targets for IS treatment extracted from **(B)**.

In order to further reveal the molecular mechanism of FFXST on IS, we conducted a topological characteristic analysis of the PPI network to determine the candidate targets of FFXST against IS. Figure 5A shows that this network consists of 704 nodes and 8,041 edges. Next, we performed the topological characteristic analysis of the combined PPI network according to the key parameters of DC and BC. After screening with DC > 45, nodes with DC were the first extracted, which included 89 nodes and 1,243 edges (Figure 5B). The candidate targets were further screened, and three targets with BC > 48 were identified (Figure 5C). Three target genes of FFXST for IS treatment were eventually identified. The information of 33 candidate targets sorted in descending order based on the value of the degree is presented in Supplementary Table S4.

### Gene ontology and kyoto encyclopedia of genes and genomes pathway enrichment analyses

**FIGURE 6:**
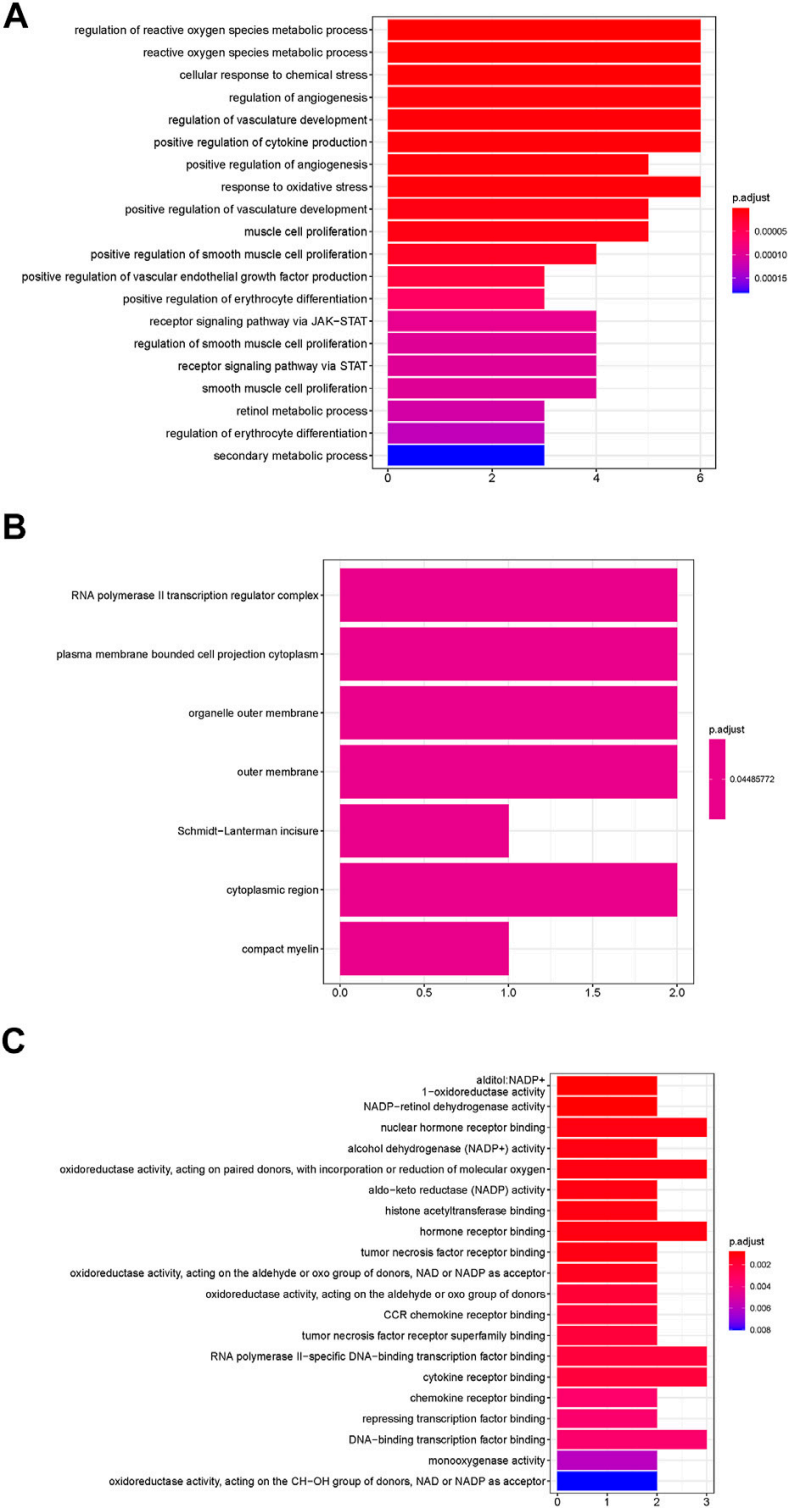
GO biological function enrichment of FFXST in the treatment of IS. **(A)** BP categories, **(B)** CC categories, and **(C)** MF categories.

**FIGURE 7:**
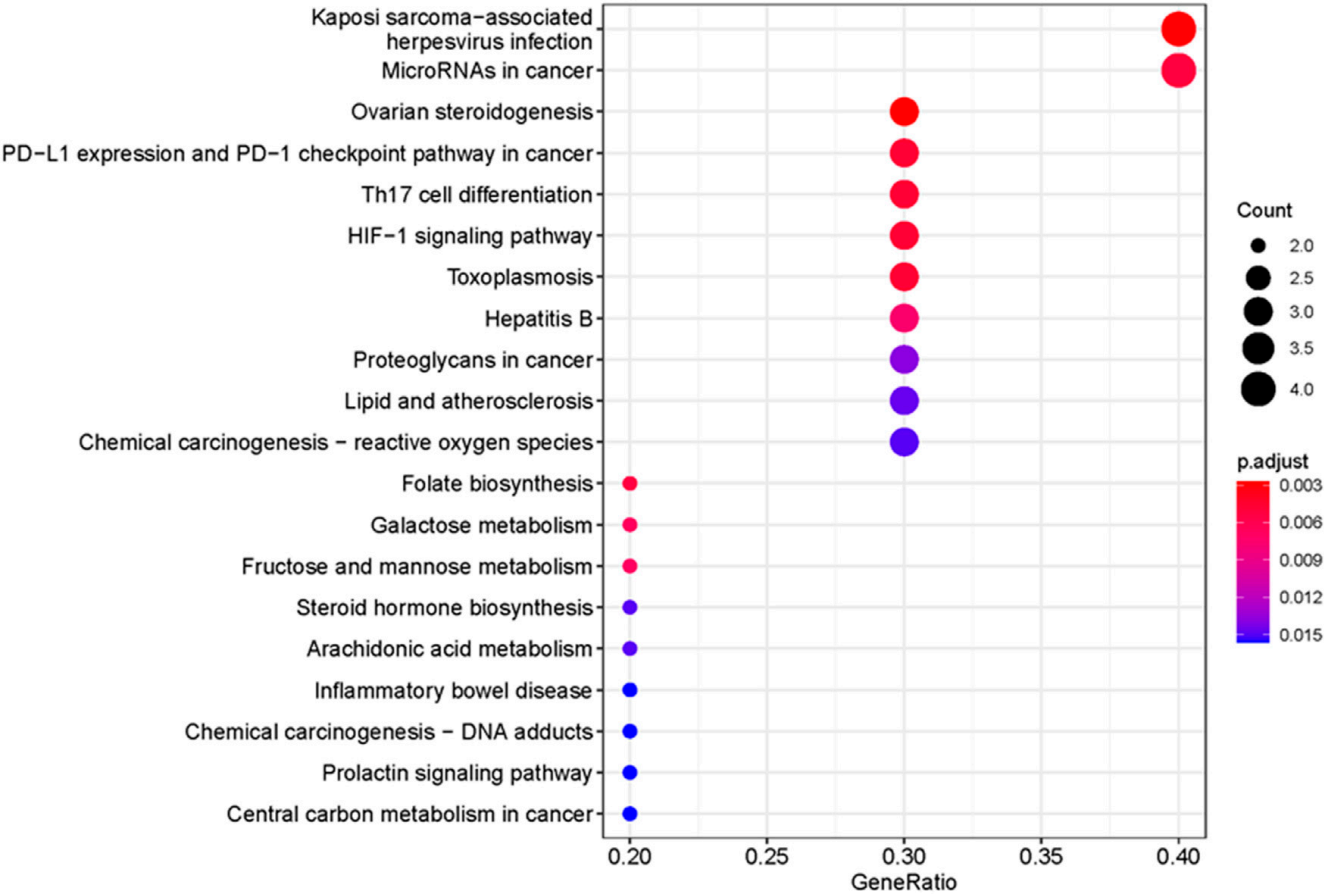
KEGG signaling pathway enrichment of FFXST in the treatment of IS. The larger the size of the dot, the more the genes are annotated in the entry, and the redder the color of the dot, the lower the q value.

**FIGURE 8:**
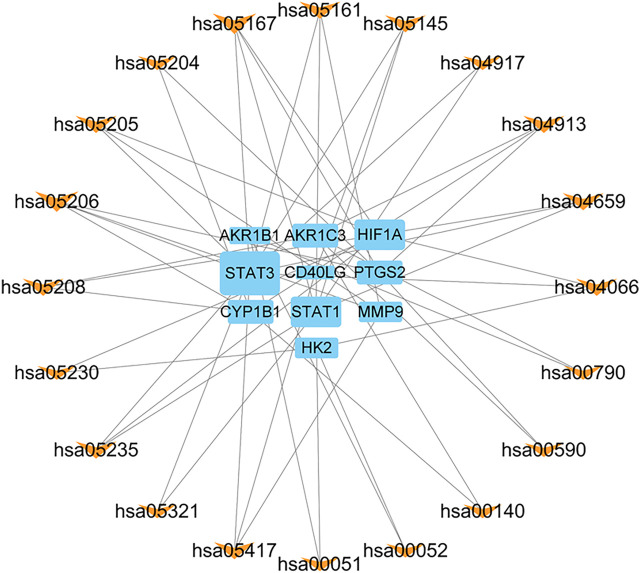
Gene–pathway network of FFXST against IS. This network shows the relationship between the enriched 20 pathways and 10 genes. The blue rectangles represent target genes, and the orange V-shapes represent pathways. The size of the graph shows the number of genes or pathways connected.

GO enrichment analysis was analyzed based on biological process (BP), cellular component (CC), and molecular function (MF). The top 20 terms are shown in Figure 6. The main enriched GO terms in BP included regulation of reactive oxygen species metabolic process, cellular response to chemical stress, regulation of angiogenesis, regulation of vasculature development, positive regulation of cytokine production, and response to oxidative stress. CC was mainly involved in RNA polymerase II transcription regulator complex, plasma membrane-bounded cell projection cytoplasm, organelle outer membrane, and outer membrane. MF was mainly involved in nuclear hormone receptor binding, oxidoreductase activity, acting on paired donors, incorporation or reduction of molecular oxygen, and hormone receptor binding. KEGG enrichment results showed that Kaposi sarcoma-associated herpesvirus infection, microRNAs in the cancer signaling pathway, Th17 cell differentiation, and HIF-1 signaling pathway were mainly influenced by FFXST in the process of treating IS (Figure 7). The genes of STAT3, STAT1, and HIF1A related to the greatest number of signaling pathways might be the key genes for FFXST against IS (Figure 8; Supplementary Table S5).

### Molecular docking

**FIGURE 9:**
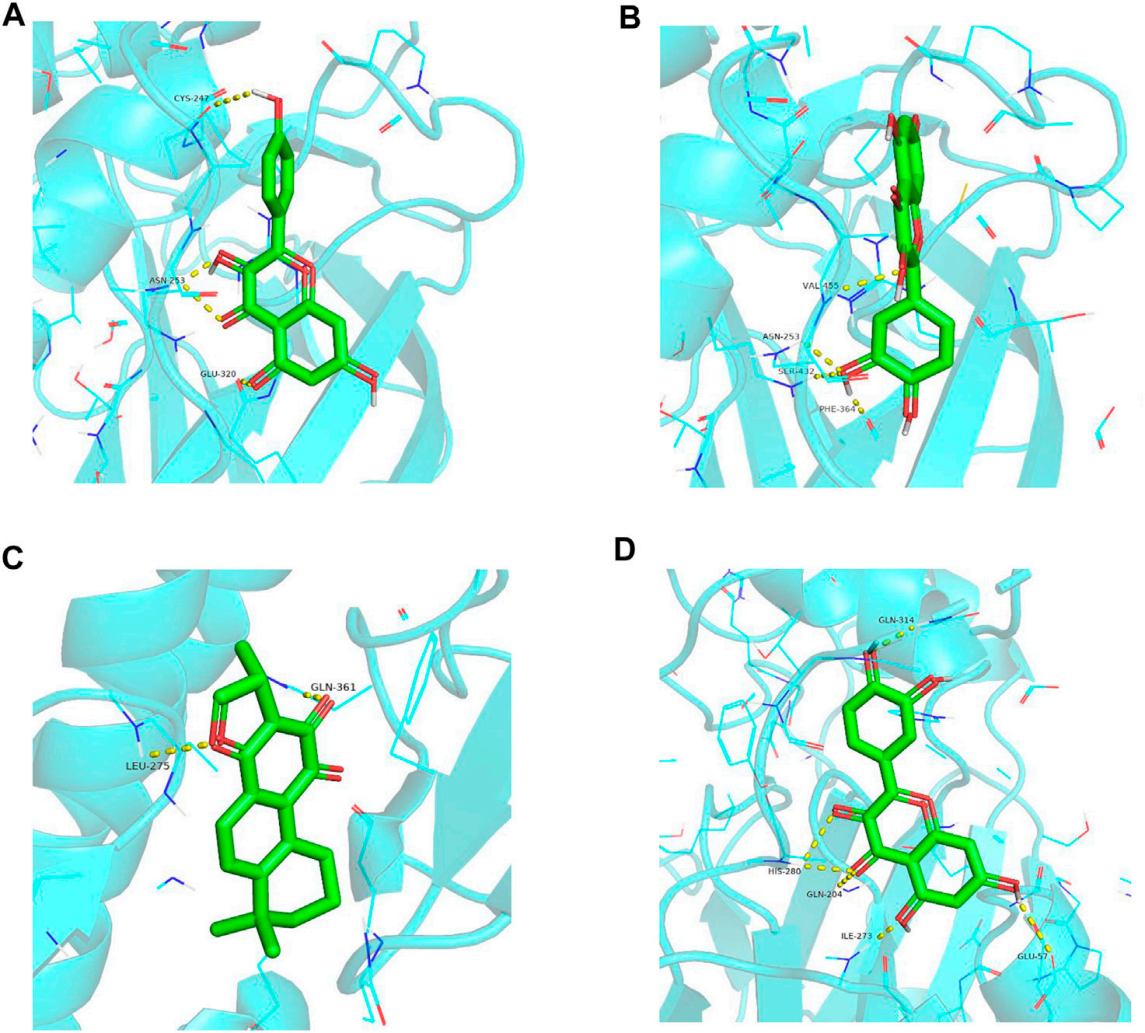
Partial diagram of molecular docking. **(A)** STAT1–kaempferol; **(B)** STAT1–quercetin; **(C)** STAT3–crytotanshinone; and **(D)** HIF1A–quercetin.

The top three core targets (STAT1, STAT3, and HIF1A) were selected from the component–target–pathway network. These core targets docking with kaempferol, cryptotanshinone, and quercetin bioactive components were verified experimentally *via* AutoDock Vina 1.1.2 software (Figure 9). The STAT3 target protein was molecularly docked with cryptotanshinone with the lowest energy value that was less than −8.7 kcal·mol^−1^(Supplementary Table S6).

## Discussion

In this study, 102 candidate compounds in the active ingredients of the four TCMs (Sanqi, Huangqi, Danshen, and Xuanshen) in FFXST play important roles in the treatment of IS and are related to a variety of molecular targets and signaling pathways, indicating that these medicinal ingredients have potent research value. Quercetin, kaempferol, and crytotanshinone were identified as the active ingredients involved in most targets, and the molecular docking outcomes also validated that they exhibit strong binding efficacy with STAT1, STAT3, and HIF1A. Quercetin is a promising natural dietary compound with preventive and therapeutic effects on a variety of diseases (Sharma et al., 2018), and its activities are embodied in antioxidant, anti-inflammatory, and pro-proliferative activities. The antioxidant and anti-inflammatory activities of quercetin in cerebral I/R treatment have been validated by various *in vivo* and *in vitro* studies. Quercetin exerts neuroprotective effects by inhibiting oxidative stress and preventing endoplasmic reticulum stress (Li et al., 2021). Kaempferol is a flavonoid compound widely present in vegetables, fruits, and Chinese herbal medicines and has various biological functions such as anti-inflammatory, antioxidant, and anti-cancer (Zhang et al., 2017). Kaempferol attenuates neuroinflammation to improve neurological deficits caused by cerebral I/R injury through the NF-κB pathway (Li et al., 2019). Cryptotanshinone, the main fat-soluble extract of *Salvia miltiorrhiza*, has shown many pharmacological activities in anti-inflammatory, antibacterial, antioxidant, anticancer, anti-ischemic/reperfusion injury, and anti-platelet aggregation (MEIm et al., 2019). Cryptotanshinone exerts neuroprotection *via* inhibiting inflammation in the cerebral I/R injury, specifically by decreasing IL-6, TNF-α, and IL-1β levels, reducing M1-type, and elevating M2-type microglia in OGD-induced BV2 cells and co-cultured microglia-neuron cells (Mao et al., 2021).

The topological analysis of the PPI network was performed for 33 intersection genes, revealing 33 strongly related proteins, among which 10 proteins (PTGS2, AKR1B1, MMP9, CD40LG, STAT3, STAT1, CYP1B1, AKR1C3, HIF1A, and HK2) are the predicted targets. Consistent with the results of the gene–pathway network, STAT3, STAT1, and HIF1A are the main targets in the PPI network with median values, suggesting that they may be the core targets of FFXST in the treatment of IS. STATs are a transcription factor family mediating cell proliferation, apoptosis, and other cellular events (Erdö et al., 2004). They are activated by free radicals, excitatory neurotransmitters, inflammatory mediators, and other cellular cytokines, during and after I/R injury (Ahn et al., 2006). The JAK/STAT signaling pathway is an important mediator involved in the regulation of neuroinflammation in the development of ischemic stroke (Deszo et al., 2004). STAT1 is activated by LPS plus IFN-γ that induces the M1 microglia polarization and accompanied by the production of inflammatory factors, such as TNF-α, IL-1β, IL-6, and iNOS (Lu et al., 2021). Phosphorylation of STAT3 modulates microglia/macrophage polarization and inhibits neuronal apoptosis and autophagy through STAT3-mediated effects in ischemic stroke (Liu et al., 2019; Tang et al., 2020; Xia et al., 2020). HIF1A regulates the expression of transcription of genes that participate in neuronal proliferation and survival after I/S injury. HIF1A can promote VEGF-mediated angiogenesis and neurogenesis under hypoxic conditions (Wu et al., 2018; Xiang et al., 2019).

To further explain clearly the mechanism of FFXST on IS treatment, we first analyzed BP, CC, and MF by GO enrichment analysis. Figure 5A shows that the BP terms of FFXST on IS were mainly related to the regulation of reactive oxygen species metabolic process, cellular response to chemical stress, regulation of angiogenesis, regulation of vasculature development, positive regulation of cytokine production, and response to oxidative stress. The oxidative stress state formed by the disruption of the homeostasis between the body’s oxidant and antioxidant systems is a key mechanism for cerebral ischemic injury. Reactive oxygen species (ROS) *in vivo* is mainly produced through pathways such as succinate dehydrogenase (SDH) and NADPH oxidase in the cellular mitochondrial succinate (Rodrigo et al., 2013; Chamorro et al., 2016). ROS acts as a key signaling molecule in the brain that directly or indirectly mediates many pathological processes of ischemic brain injury. It was also demonstrated that FFXST affects specific CC and MF terms, including RNA polymerase II transcription regulator complex, plasma membrane-bounded cell projection cytoplasm, organelle outer membrane, nuclear hormone receptor binding, oxidoreductase activity, acting on paired donors, with incorporation or reduction of molecular oxygen, and hormone receptor binding. KEGG pathways involving Kaposi sarcoma-associated herpesvirus infection, microRNAs in the cancer signaling pathway, Th17 cell differentiation, and HIF-1 signaling pathway were significantly enriched.

The main cells infected by Kaposi sarcoma-associated herpesvirus infection (KSHV) are epithelial cells, endothelial cells, B cells, and macrophages. KSHV infection of cells leads to increased synthesis and secretion of cytokines, which promotes cell proliferation and differentiation, and its infection is accompanied by pathological changes in the nervous system (Tso et al., 2017). MicroRNAs (miRNAs) are widely involved in the development of ischemic stroke, which can assist in the early diagnosis of the disease and possibly evaluate the prognosis (Xu et al., 2018). The research study has found that overexpression of miR-98 reduces cerebral infarct size in tMCAO mice, reduces the infiltration of pro-inflammatory Ly6Chi leukocytes, attenuates BBB permeability, and improves motility in mice with cerebral ischemia dysfunction (Bernstein et al., 2020). Therefore, the study of specific miRNAs related to ischemic stroke can make it a drug target for the treatment of ischemic stroke and bring new hope to the treatment of human ischemic stroke. Immune inflammatory response plays a key role in the pathophysiological process of acute ischemic stroke. T helper 17 (Th17) cells and regulatory T (Treg) cells are two important immune cells derived from CD4^+^ T cells. Th17/Treg balance is involved in the inflammatory response and plays an important role in immune regulation (Dolati et al., 2018). Th17 cells recruit and activate neutrophils mainly through the cytokines they secrete, stimulate epithelial cells to produce a defensive effect, and mediate inflammatory responses. It can secrete TNF-α, IL-6, IL-17A, IL-17F, IL-21, and IL-22, etc (Romagnani et al., 2009; Guo et al., 2018). Among them, IL-17A participates in the proliferation, maturation, and chemotaxis of neutrophils and regulates neutrophil apoptosis, promotes the maturation and chemotactic process of dendritic cells, and plays a synergistic role in stimulating the activation of T cells (Komiyama et al., 2006). Therefore, FFXST may help to prevent and treat IS by maintaining Th17/Treg balance to regulate the immune inflammatory response. The HIF-1 signaling pathway was also significantly enriched in this study, which suggested that the regulation of the HIF-1 signaling pathway might be one of the mechanisms of FFXST for the treatment of IS. HIF-1 is a nuclear transcriptional regulator found in response to hypoxia in mammalian cells. By inducing the expression of target genes during hypoxia and regulating anaerobic metabolism, angiogenesis, and the increase of erythropoietin, etc., the hypoxic tissue cells maintain a certain oxygen concentration and enable cells to survive in a state of hypoxia (Pan et al., 2021). The study found that the expression of HIF-1α and its target genes VEGF, EPO, and GTP in the ischemic penumbra increased significantly after ischemia and hypoxia (Stowe et al., 2008; Zhang et al., 2010), which was conducive to promoting collateral angiogenesis and glucose metabolism, improving the blood flow supply and energy supply in the penumbra, and enabling the survival of this part of neurons. In addition, FFXST may function by interfering with other pathways, including ovarian steroidogenesis, PD-L1 expression, and PD-1 checkpoint pathway in cancer, toxoplasmosis, and hepatitis B.

The potential mechanism and targets of FFXST for IS were investigated using network pharmacology and molecular docking in this study. Quercetin, kaempferol, and crytotanshinone were identified as the active ingredients associated with most targets. FFXST may exert anti-IS function and the regulation of pathways including Kaposi sarcoma-associated herpesvirus infection, microRNAs in the cancer signaling pathway, Th17 cell differentiation, and HIF-1 signaling pathway. STAT1, STAT3, and HIF1A were the important targets of FFXST in the treatment of IS. This research initially explored the active components, core targets, and signaling pathways of FFXST in the treatment of IS, and the active components and core targets were further verified by means of molecular docking. This study systematically illustrated the characteristics of multi-component, multi-target, and multi-pathway of FFXST for IS. We demonstrated that FFXST protection against IS may relate to the regulation of oxidative stress, immune inflammatory response, and angiogenesis through the relevant signaling pathways. However, due to the complexity of TCM ingredients, the preliminary exploration of FFXST based on network pharmacology is still insufficient. Aiming at the molecular targets and signaling pathway of FFXST for IS in the prediction results, further experimental verification will be conducted in the later stage, in order to provide a theoretical basis and reference for the pharmacological study of the pharmacodynamic components of FFXST in the treatment of IS and provide a basis for strengthening the optimization of experimental design and further discussion in the later stage.

## Data Availability

The datasets presented in this study can be found in online repositories. The names of the repository/repositories and accession number(s) can be found in the article/Supplementary Material.

## References

[B1] Adams H. P. Jr. del Zoppo G. Alberts M. J. Bhatt D. L. Brass L. Furlan A. (2007). Guidelines for the early management of adults with ischemic stroke: A guideline from the American heart association/American stroke association stroke council, clinical cardiology council, cardiovascular radiology and intervention council, and the atherosclerotic peripheral vascular disease and quality of care outcomes in research interdisciplinary working groups: The American Academy of neurology affirms the value of this guideline as an educational tool for neurologists. Circulation 115 (20), e478–534. 10.1161/circulationaha.107.181486 17515473

[B2] Ahn Y. H. Lee G. Kang S. K. (2006). Molecular insights of the injured lesions of rat spinal cords: Inflammation, apoptosis, and cell survival. Biochem. Biophys. Res. Commun. 348 (2), 560–570. 10.1016/j.bbrc.2006.07.105 16890196

[B3] Bernstein D. L. Zuluaga-Ramirez V. Gajghate S. Reichenbach N. L. Polyak B. Persidsky Y. (2020). miR-98 reduces endothelial dysfunction by protecting blood-brain barrier (BBB) and improves neurological outcomes in mouse ischemia/reperfusion stroke model. J. Cereb. Blood Flow. Metab. 40 (10), 1953–1965. 10.1177/0271678x19882264 31601141 PMC7786850

[B4] Campbell B. C. V. De Silva D. A. Macleod M. R. Coutts S. B. Schwamm L. H. Davis S. M. (2019). Ischaemic stroke. Nat. Rev. Dis. Prim. 5 (1), 70. 10.1038/s41572-019-0118-8 31601801

[B5] Chamorro Á. Dirnagl U. Urra X. Planas A. M. (2016). Neuroprotection in acute stroke: Targeting excitotoxicity, oxidative and nitrosative stress, and inflammation. Lancet. Neurol. 15 (8), 869–881. 10.1016/s1474-4422(16)00114-9 27180033

[B6] Deszo E. L. Brake D. K. Kelley K. W. Freund G. G. (2004). IL-4-dependent CD86 expression requires JAK/STAT6 activation and is negatively regulated by PKCdelta. Cell. Signal. 16 (2), 271–280. 10.1016/s0898-6568(03)00137-2 14636897

[B7] Dibajnia P. Morshead C. M. (2013). Role of neural precursor cells in promoting repair following stroke. Acta Pharmacol. Sin. 34 (1), 78–90. 10.1038/aps.2012.107 23064725 PMC4086492

[B8] Dirnagl U. Endres M. (2014). Found in translation: Preclinical stroke research predicts human pathophysiology, clinical phenotypes, and therapeutic outcomes. Stroke 45 (5), 1510–1518. 10.1161/strokeaha.113.004075 24652307

[B9] Dolati S. Ahmadi M. Khalili M. Taheraghdam A. A. Siahmansouri H. Babaloo Z. (2018). Peripheral Th17/Treg imbalance in elderly patients with ischemic stroke. Neurol. Sci. 39 (4), 647–654. 10.1007/s10072-018-3250-4 29353353

[B10] Erdö F. Trapp T. Mies G. Hossmann K. A. (2004). Immunohistochemical analysis of protein expression after middle cerebral artery occlusion in mice. Acta Neuropathol. 107 (2), 127–136. 10.1007/s00401-003-0789-8 14648078

[B11] Guo Y. Chen X. Li D. Liu H. Ding Y. Han R. (2018). PR-957 mediates neuroprotection by inhibiting Th17 differentiation and modulating cytokine production in a mouse model of ischaemic stroke. Clin. Exp. Immunol. 193 (2), 194–206. 10.1111/cei.13132 29603201 PMC6046491

[B12] Jiang Y. Liu N. Zhu S. Hu X. Chang D. Liu J. (2019). Elucidation of the mechanisms and molecular targets of yiqi shexue formula for treatment of primary immune thrombocytopenia based on network pharmacology. Front. Pharmacol. 10, 1136. 10.3389/fphar.2019.01136 31632275 PMC6780007

[B13] Kang X. Su S. Hong W. Geng W. Tang H. (2021). Research progress on the ability of astragaloside IV to protect the brain against ischemia-reperfusion injury. Front. Neurosci. 15, 755902. 10.3389/fnins.2021.755902 34867166 PMC8637115

[B14] Komiyama Y. Nakae S. Matsuki T. Nambu A. Ishigame H. Kakuta S. (2006). IL-17 plays an important role in the development of experimental autoimmune encephalomyelitis. J. Immunol. 177 (1), 566–573. 10.4049/jimmunol.177.1.566 16785554

[B15] Li D. H. Su Y. F. Sun C. X. Fan H. F. Gao W. J. (2020). A network pharmacology-based identification study on the mechanism of xiao-xu-ming decoction for cerebral ischemic stroke. Evid. Based. Complement. Altern. Med. 2020, 2507074. 10.1155/2020/2507074 PMC7593742 33133212

[B16] Li J. Zhao P. Li Y. Tian Y. Wang Y. (2015). Systems pharmacology-based dissection of mechanisms of Chinese medicinal formula Bufei Yishen as an effective treatment for chronic obstructive pulmonary disease. Sci. Rep. 5, 15290. 10.1038/srep15290 26469778 PMC4606809

[B17] Li M. T. Ke J. Guo S. F. Wu Y. Bian Y. F. Shan L. L. (2021). The protective effect of quercetin on endothelial cells injured by hypoxia and reoxygenation. Front. Pharmacol. 12, 732874. 10.3389/fphar.2021.732874 34744717 PMC8564287

[B18] Li W. H. Cheng X. Yang Y. L. Liu M. Zhang S. S. Wang Y. H. (2019). Kaempferol attenuates neuroinflammation and blood brain barrier dysfunction to improve neurological deficits in cerebral ischemia/reperfusion rats. Brain Res. 1722, 146361. 10.1016/j.brainres.2019.146361 31377105

[B19] Liu Z. J. Ran Y. Y. Qie S. Y. Gong W. J. Gao F. H. Ding Z. T. (2019). Melatonin protects against ischemic stroke by modulating microglia/macrophage polarization toward anti-inflammatory phenotype through STAT3 pathway. CNS Neurosci. Ther. 25 (12), 1353–1362. 10.1111/cns.13261 31793209 PMC6887673

[B20] Lu Y. Zhou M. Li Y. Li Y. Hua Y. Fan Y. (2021). Minocycline promotes functional recovery in ischemic stroke by modulating microglia polarization through STAT1/STAT6 pathways. Biochem. Pharmacol. 186, 114464. 10.1016/j.bcp.2021.114464 33577892

[B21] Mao Y. Qu Y. Wang Q. (2021). Cryptotanshinone reduces neurotoxicity induced by cerebral ischemia-reperfusion injury involving modulation of microglial polarization. Restor. Neurol. Neurosci. 39 (3), 209–220. 10.3233/rnn-201070 34219678

[B22] MEIm X. D. Cao Y. F. Che Y. Y. Li J. Shang Z. P. Zhao W. J. (2019). Danshen: A phytochemical and pharmacological overview. Chin. J. Nat. Med. 17 (1), 59–80. 10.1016/s1875-5364(19)30010-x 30704625

[B23] Pan Z. Ma G. Kong L. Du G. (2021). Hypoxia-inducible factor-1: Regulatory mechanisms and drug development in stroke. Pharmacol. Res. 170, 105742. 10.1016/j.phrs.2021.105742 34182129

[B24] Papanagiotou P. Ntaios G. (2018). Endovascular thrombectomy in acute ischemic stroke. Circ. Cardiovasc. Interv. 11 (1), e005362. 10.1161/circinterventions.117.005362 29311286

[B25] Rodrigo R. Fernández-Gajardo R. Gutiérrez R. Matamala J. M. Carrasco R. Miranda-Merchak A. (2013). Oxidative stress and pathophysiology of ischemic stroke: Novel therapeutic opportunities. CNS Neurol. Disord. Drug Targets 12 (5), 698–714. 10.2174/1871527311312050015 23469845

[B26] Romagnani S. Maggi E. Liotta F. Cosmi L. Annunziato F. (2009). Properties and origin of human Th17 cells. Mol. Immunol. 47 (1), 3–7. 10.1016/j.molimm.2008.12.019 19193443

[B27] Sharma A. Kashyap D. Sak K. Tuli H. S. Sharma A. K. (2018). Therapeutic charm of quercetin and its derivatives: A review of research and patents. Pharm. Pat. Anal. 7 (1), 15–32. 10.4155/ppa-2017-0030 29227203

[B28] Stowe A. M. Plautz E. J. Nguyen P. Frost S. B. Eisner-Janowicz I. Barbay S. (2008). Neuronal HIF-1 alpha protein and VEGFR-2 immunoreactivity in functionally related motor areas following a focal M1 infarct. J. Cereb. Blood Flow. Metab. 28 (3), 612–620. 10.1038/sj.jcbfm.9600560 17895908 PMC3232012

[B29] Tang H. Gamdzyk M. Huang L. Gao L. Lenahan C. Kang R. (2020). Delayed recanalization after MCAO ameliorates ischemic stroke by inhibiting apoptosis via HGF/c-Met/STAT3/Bcl-2 pathway in rats. Exp. Neurol. 330, 113359. 10.1016/j.expneurol.2020.113359 32428505

[B30] Tso F. Y. Sawyer A. Kwon E. H. Mudenda V. Langford D. Zhou Y. (2017). Kaposi's sarcoma-associated herpesvirus infection of neurons in HIV-positive patients. J. Infect. Dis. 215 (12), 1898–1907. 10.1093/infdis/jiw545 27932611 PMC5853869

[B31] Wang K. Lei L. Cao J. Qiao Y. Liang R. Duan J. (2021). Network pharmacology-based prediction of the active compounds and mechanism of Buyang Huanwu Decoction for ischemic stroke. Exp. Ther. Med. 22 (4), 1050. 10.3892/etm.2021.10484 34434264 PMC8353622

[B32] Wang L. Zhu T. Xu H. B. Pu X. P. Zhao X. Tian F. (2021). Effects of notoginseng leaf triterpenes on small molecule metabolism after cerebral ischemia/reperfusion injury assessed using MALDI-MS imaging. Ann. Transl. Med. 9 (3), 246. 10.21037/atm-20-4898 33708873 PMC7940900

[B33] Wu B. Liu M. Zhang S. (2007). Dan Shen agents for acute ischaemic stroke. Cochrane Database Syst. Rev. 38 (2), CD004295. 10.1002/14651858.CD004295.pub3 PMC12832167 17443544

[B34] Wu X. Liu S. Hu Z. Zhu G. Zheng G. Wang G. (2018). Enriched housing promotes post-stroke neurogenesis through calpain 1-STAT3/HIF-1α/VEGF signaling. Brain Res. Bull. 139, 133–143. 10.1016/j.brainresbull.2018.02.018 29477834

[B35] Xia Y. Ling X. Hu G. Zhu Q. Zhang J. Li Q. (2020). Small extracellular vesicles secreted by human iPSC-derived MSC enhance angiogenesis through inhibiting STAT3-dependent autophagy in ischemic stroke. Stem Cell Res. Ther. 11 (1), 313. 10.1186/s13287-020-01834-0 32698909 PMC7374834

[B36] Xiang Y. Yao X. Wang X. Zhao H. Zou H. Wang L. (2019). Houshiheisan promotes angiogenesis via HIF-1α/VEGF and SDF-1/CXCR4 pathways: *In vivo* and *in vitro* . Biosci. Rep. 39 (10), BSR20191006. 10.1042/bsr20191006 31652450 PMC6822506

[B37] Xu W. Gao L. Zheng J. Li T. Shao A. Reis C. (2018). The roles of MicroRNAs in stroke: Possible therapeutic targets. Cell Transpl. 27 (12), 1778–1788. 10.1177/0963689718773361 PMC6300776 29871520

[B38] Zeng L. Yang K. Liu H. Zhang G. (2017). A network pharmacology approach to investigate the pharmacological effects of Guizhi Fuling Wan on uterine fibroids. Exp. Ther. Med. 14 (5), 4697–4710. 10.3892/etm.2017.5170 29201170 PMC5704263

[B39] Zhang X. Deguchi K. Yamashita T. Ohta Y. Shang J. Tian F. (2010). Temporal and spatial differences of multiple protein expression in the ischemic penumbra after transient MCAO in rats. Brain Res. 1343, 143–152. 10.1016/j.brainres.2010.04.027 20417628

[B40] Zhang Y. W. Shao D. Y. Shi J. L. Zhu J. Huang Q. S. Yang H. (2017). A review on biological activities of kaempferol. Chin. Bull. Life Sci 29 (4), 400–405. 10.13376/j.cbls/2017053

[B41] Zhong Y. Fang Y. (2020). Exploring the pharmacological mechanism of cassiae semen a cting on cataracts based on a network pharmacology approach.

[B42] Zhou Z. Lu J. Liu W. W. Manaenko A. Hou X. Mei Q. (2018). Advances in stroke pharmacology. Pharmacol. Ther. 191, 23–42. 10.1016/j.pharmthera.2018.05.012 29807056

[B43] Zhu T. Wan Q. (2022). Pharmacological properties and mechanisms of notoginsenoside R1 in ischemia-reperfusion injury: A mini-review. Chin. J. Traumatology. 10.1016/j.cjtee.2022.06.008 PMC9912185 35922249

[B44] Zhu T. Wang L. Feng Y. Sun G. Sun X. (2021a). Classical active ingredients and extracts of Chinese herbal medicines: Pharmacokinetics, pharmacodynamics, and molecular mechanisms for ischemic stroke. Oxid. Med. Cell. Longev. 2021, 8868941. 10.1155/2021/8868941 33791075 PMC7984881

[B45] Zhu T. Wang L. Tian F. Zhao X. Pu X. P. Sun G. B. (2020). Anti-ischemia/reperfusion injury effects of notoginsenoside R1 on small molecule metabolism in rat brain after ischemic stroke as visualized by MALDI-MS imaging. Biomed. Pharmacother. 129, 110470. 10.1016/j.biopha.2020.110470 32768957

[B46] Zhu T. Wang L. Wang L. P. Wan Q. (2022). Therapeutic targets of neuroprotection and neurorestoration in ischemic stroke: Applications for natural compounds from medicinal herbs. Biomed. Pharmacother. 148, 112719. 10.1016/j.biopha.2022.112719 35168073

[B47] Zhu T. Xie W. J. Wang L. Jin X. B. Meng X. B. Sun G. B. (2021b). Notoginsenoside R1 activates the NAMPT-NAD(+)-SIRT1 cascade to promote postischemic angiogenesis by modulating Notch signaling. Biomed. Pharmacother. 140, 111693. 10.1016/j.biopha.2021.111693 34029951

