# Supplementary material for: Investigation into the potential mechanism and molecular targets of Fufang Xueshuantong capsule for the treatment of ischemic stroke based on network pharmacology and molecular docking
Source: Front Pharmacol. 2022 Sep 15;13:949644. doi: 10.3389/fphar.2022.949644 (PMC9524248; doi:10.3389/fphar.2022.949644)
Supplement: Supplementary file 1 [file Table1.DOCX]

| **SUPPLEMENTARY TABLE 1** Characteristics of active ingredients in FFXST. | | | | |
| --- | --- | --- | --- | --- |
| Ingredients | Molecule ID | Molecule name | OB (%) | DL |
| Sanqi | MOL001494 | Mandenol | 42.00 | 0.19 |
| Sanqi | MOL001792 | DFV | 32.76 | 0.18 |
| Sanqi | MOL002879 | Diop | 43.59 | 0.39 |
| Sanqi | MOL000358 | Beta-sitosterol | 36.91 | 0.75 |
| Sanqi | MOL000449 | Stigmasterol | 43.83 | 0.76 |
| Sanqi | MOL005344 | Ginsenoside rh2 | 36.32 | 0.56 |
| Sanqi | MOL007475 | Ginsenoside f2 | 36.43 | 0.25 |
| Sanqi | MOL000098 | Quercetin | 46.43 | 0.28 |
| Huangqi | MOL000211 | Mairin | 55.38 | 0.78 |
| Huangqi | MOL000239 | Jaranol | 50.83 | 0.29 |
| Huangqi | MOL000296 | Hederagenin | 36.91 | 0.75 |
| Huangqi | MOL000033 | (3S,8S,9S,10R,13R,14S,17R)-10,13-dimethyl-17-[(2R,5S)-5-propan-2-yloctan-2-yl]-2,3,4,7,8,9,11,12,14,15,16,17-dodecahydro-1H-cyclopenta[a]phenanthren-3-ol | 36.23 | 0.78 |
| Huangqi | MOL000354 | Isorhamnetin | 49.60 | 0.31 |
| Huangqi | MOL000371 | 3,9-di-O-methylnissolin | 53.74 | 0.48 |
| Huangqi | MOL000374 | 5'-hydroxyiso-muronulatol-2',5'-di-O-glucoside | 41.72 | 0.69 |
| Huangqi | MOL000378 | 7-O-methylisomucronulatol | 74.69 | 0.30 |
| Huangqi | MOL000379 | 9,10-dimethoxypterocarpan-3-O-β-D-glucoside | 36.74 | 0.92 |
| Huangqi | MOL000380 | (6aR,11aR)-9,10-dimethoxy-6a,11a-dihydro-6H-benzofurano[3,2-c]chromen-3-ol | 64.26 | 0.42 |
| Huangqi | MOL000387 | Bifendate | 31.10 | 0.67 |
| Huangqi | MOL000392 | Formononetin | 69.67 | 0.21 |
| Huangqi | MOL000398 | Isoflavanone | 109.99 | 0.30 |
| Huangqi | MOL000417 | Calycosin | 47.75 | 0.24 |
| Huangqi | MOL000422 | Kaempferol | 41.88 | 0.24 |
| Huangqi | MOL000433 | FA | 68.96 | 0.71 |
| Huangqi | MOL000438 | (3R)-3-(2-hydroxy-3,4-dimethoxyphenyl)chroman-7-ol | 67.67 | 0.26 |
| Huangqi | MOL000439 | Isomucronulatol-7,2'-di-O-glucosiole | 49.28 | 0.62 |
| Huangqi | MOL000442 | 1,7-Dihydroxy-3,9-dimethoxy pterocarpene | 39.05 | 0.48 |
| Huangqi | MOL000098 | Quercetin | 46.43 | 0.28 |
| Danshen | MOL001601 | 1,2,5,6-tetrahydrotanshinone | 38.75 | 0.36 |
| Danshen | MOL001659 | Poriferasterol | 43.83 | 0.76 |
| Danshen | MOL001771 | Poriferast-5-en-3beta-ol | 36.91 | 0.75 |
| Danshen | MOL001942 | Isoimperatorin | 45.46 | 0.23 |
| Danshen | MOL002222 | Sugiol | 36.11 | 0.28 |
| Danshen | MOL002651 | Dehydrotanshinone II A | 43.76 | 0.4 |
| Danshen | MOL002776 | Baicalin | 40.12 | 0.75 |
| Danshen | MOL000569 | Digallate | 61.85 | 0.26 |
| Danshen | MOL000006 | Luteolin | 36.16 | 0.25 |
| Danshen | MOL006824 | α-amyrin | 39.51 | 0.76 |
| Danshen | MOL007036 | 5,6-dihydroxy-7-isopropyl-1,1-dimethyl-2,3-dihydrophenanthren-4-one | 33.77 | 0.29 |
| Danshen | MOL007041 | 2-isopropyl-8-methylphenanthrene-3,4-dione | 40.86 | 0.23 |
| Danshen | MOL007045 | 3α-hydroxytanshinoneⅡa | 44.93 | 0.44 |
| Danshen | MOL007048 | (E)-3-[2-(3,4-dihydroxyphenyl)-7-hydroxy-benzofuran-4-yl]acrylic acid | 48.24 | 0.31 |
| Danshen | MOL007049 | 4-methylenemiltirone | 34.35 | 0.23 |
| Danshen | MOL007050 | 2-(4-hydroxy-3-methoxyphenyl)-5-(3-hydroxypropyl)-7-methoxy-3-benzofurancarboxaldehyde | 62.78 | 0.4 |
| Danshen | MOL007051 | 6-o-syringyl-8-o-acetyl shanzhiside methyl ester | 46.69 | 0.71 |
| Danshen | MOL007058 | Formyltanshinone | 73.44 | 0.42 |
| Danshen | MOL007059 | 3-beta-Hydroxymethyllenetanshiquinone | 32.16 | 0.41 |
| Danshen | MOL007061 | Methylenetanshinquinone | 37.07 | 0.36 |
| Danshen | MOL007063 | Przewalskin a | 37.11 | 0.65 |
| Danshen | MOL007064 | Przewalskin b | 110.32 | 0.44 |
| Danshen | MOL007068 | Przewaquinone B | 62.24 | 0.41 |
| Danshen | MOL007069 | Przewaquinone c | 55.74 | 0.4 |
| Danshen | MOL007070 | (6S,7R)-6,7-dihydroxy-1,6-dimethyl-8,9-dihydro-7H-naphtho[8,7-g]benzofuran-10,11-dione | 41.31 | 0.45 |
| Danshen | MOL007071 | Przewaquinone f | 40.31 | 0.46 |
| Danshen | MOL007077 | Sclareol | 43.67 | 0.21 |
| Danshen | MOL007079 | Tanshinaldehyde | 52.47 | 0.45 |
| Danshen | MOL007081 | Danshenol B | 57.95 | 0.56 |
| Danshen | MOL007082 | Danshenol A | 56.97 | 0.52 |
| Danshen | MOL007085 | Salvilenone | 30.38 | 0.38 |
| Danshen | MOL007088 | Cryptotanshinone | 52.34 | 0.4 |
| Danshen | MOL007093 | Dan-shexinkum d | 38.88 | 0.55 |
| Danshen | MOL007094 | Danshenspiroketallactone | 50.43 | 0.31 |
| Danshen | MOL007098 | Deoxyneocryptotanshinone | 49.4 | 0.29 |
| Danshen | MOL007100 | Dihydrotanshinlactone | 38.68 | 0.32 |
| Danshen | MOL007101 | Dihydrotanshinone Ⅰ | 45.04 | 0.36 |
| Danshen | MOL007105 | Epidanshenspiroketallactone | 68.27 | 0.31 |
| Danshen | MOL007107 | C09092 | 36.07 | 0.25 |
| Danshen | MOL007108 | Isocryptotanshi-none | 54.98 | 0.39 |
| Danshen | MOL007111 | Isotanshinone II | 49.92 | 0.4 |
| Danshen | MOL007115 | Manool | 45.04 | 0.2 |
| Danshen | MOL007118 | Microstegiol | 39.61 | 0.28 |
| Danshen | MOL007119 | Miltionone Ⅰ | 49.68 | 0.32 |
| Danshen | MOL007120 | Miltionone Ⅱ | 71.03 | 0.44 |
| Danshen | MOL007121 | Miltipolone | 36.56 | 0.37 |
| Danshen | MOL007122 | Miltirone | 38.76 | 0.25 |
| Danshen | MOL007123 | Miltirone Ⅱ | 44.95 | 0.24 |
| Danshen | MOL007124 | Neocryptotanshinone ii | 39.46 | 0.23 |
| Danshen | MOL007125 | Neocryptotanshinone | 52.49 | 0.32 |
| Danshen | MOL007127 | 1-methyl-8,9-dihydro-7H-naphtho[5,6-g]benzofuran-6,10,11-trione | 34.72 | 0.37 |
| Danshen | MOL007130 | Prolithospermic acid | 64.37 | 0.31 |
| Danshen | MOL007132 | (2R)-3-(3,4-dihydroxyphenyl)-2-[(Z)-3-(3,4-dihydroxyphenyl)acryloyl]oxy-propionic acid | 109.38 | 0.35 |
| Danshen | MOL007140 | (Z)-3-[2-[(E)-2-(3,4-dihydroxyphenyl)vinyl]-3,4-dihydroxy-phenyl]acrylic acid | 88.54 | 0.26 |
| Danshen | MOL007141 | Salvianolic acid g | 45.56 | 0.61 |
| Danshen | MOL007142 | Salvianolic acid j | 43.38 | 0.72 |
| Danshen | MOL007143 | Salvilenone Ⅰ | 32.43 | 0.23 |
| Danshen | MOL007145 | Salviolone | 31.72 | 0.24 |
| Danshen | MOL007149 | NSC 122421 | 34.49 | 0.28 |
| Danshen | MOL007150 | (6S)-6-hydroxy-1-methyl-6-methylol-8,9-dihydro-7H-naphtho[8,7-g]benzofuran-10,11-quinone | 75.39 | 0.46 |
| Danshen | MOL007151 | Tanshindiol B | 42.67 | 0.45 |
| Danshen | MOL007152 | Przewaquinone E | 42.85 | 0.45 |
| Danshen | MOL007154 | Tanshinone iia | 49.89 | 0.4 |
| Danshen | MOL007155 | (6S)-6-(hydroxymethyl)-1,6-dimethyl-8,9-dihydro-7H-naphtho[8,7-g]benzofuran-10,11-dione | 65.26 | 0.45 |
| Danshen | MOL007156 | Tanshinone Ⅵ | 45.64 | 0.30 |
| Xuanshen | MOL001925 | Paeoniflorin_qt | 68.18 | 0.4 |
| Xuanshen | MOL002222 | Sugiol | 36.11 | 0.28 |
| Xuanshen | MOL000358 | Beta-sitosterol | 36.91 | 0.75 |
| Xuanshen | MOL000359 | Sitosterol | 36.91 | 0.75 |
| Xuanshen | MOL007657 | Scropolioside A_qt | 38.63 | 0.77 |
| Xuanshen | MOL007658 | 14-deoxy-12(R)-sulfoandrographolide | 62.57 | 0.42 |
| Xuanshen | MOL007659 | Scropolioside D | 36.62 | 0.4 |
| Xuanshen | MOL007660 | Scropolioside D_qt | 33.17 | 0.82 |
| Xuanshen | MOL007662 | Harpagoside_qt | 122.87 | 0.32 |
