# Supplementary material for: Investigation into the potential mechanism and molecular targets of Fufang Xueshuantong capsule for the treatment of ischemic stroke based on network pharmacology and molecular docking
Source: Front Pharmacol. 2022 Sep 15;13:949644. doi: 10.3389/fphar.2022.949644 (PMC9524248; doi:10.3389/fphar.2022.949644)
Supplement: Supplementary file 2 [file Table2.DOCX]

**SUPPLEMENTARY TABLE 2** The top 20 up-regulated genes and down-regulated genes.

| Gene names | LogFC | P value | Regulation direction |
| --- | --- | --- | --- |
| ARG1 | 1.679861016 | 1.62E-09 | up |
| MMP9 | 1.350327617 | 2.36E-07 | up |
| HLA-DRB1 | 1.197213295 | 0.022079818 | up |
| ORM1 | 1.1449205 | 0.000693573 | up |
| S100A12 | 1.143601128 | 2.25E-06 | up |
| CA4 | 1.095595076 | 4.53E-06 | up |
| LY96 | 1.077518441 | 0.000359503 | up |
| APOBEC3A | 1.076937006 | 0.000371451 | up |
| CRISPLD2 | 1.058302138 | 2.67E-08 | up |
| FCGR3B | 1.019643667 | 0.000332904 | up |
| RIS1 | 1.014191078 | 0.003475081 | up |
| FTHL11 | 0.995260641 | 0.00346375 | up |
| ACSL1 | 0.957983862 | 9.16E-06 | up |
| PADI4 | 0.951805015 | 1.31E-07 | up |
| CSPG2 | 0.941442888 | 2.48E-06 | up |
| FOLR3 | 0.937548645 | 0.000147939 | up |
| FTHL3 | 0.933579984 | 0.002234266 | up |
| SDPR | 0.919448733 | 0.000134015 | up |
| OLFM4 | 0.91554037 | 0.007545193 | up |
| PDK4 | 0.90464469 | 4.99E-09 | up |
| CCR7 | -1.371623175 | 3.57E-07 | down |
| VPREB3 | -1.25346473 | 3.31E-07 | down |
| HLA-DQB1 | -1.164297433 | 3.20E-05 | down |
| MAL | -1.149061957 | 1.16E-09 | down |
| CD6 | -1.084021225 | 8.06E-10 | down |
| IL7R | -1.074726583 | 6.30E-08 | down |
| C16orf30 | -1.072343749 | 1.01E-09 | down |
| PASK | -1.068014865 | 5.72E-08 | down |
| NELL2 | -1.048066472 | 1.17E-06 | down |
| ID3 | -1.046217351 | 2.16E-11 | down |
| ITK | -1.033865176 | 1.28E-09 | down |
| LEF1 | -1.017400479 | 4.97E-07 | down |
| GZMK | -1.009452297 | 7.59E-05 | down |
| FAIM3 | -1.000471219 | 2.95E-08 | down |
| CD79B | -0.989665283 | 8.71E-08 | down |
| FLT3LG | -0.95574612 | 4.76E-10 | down |
| FCRLM1 | -0.946343661 | 3.49E-05 | down |
| MFGE8 | -0.934449228 | 5.59E-08 | down |
| FAM113B | -0.930009418 | 2.13E-08 | down |
| ECHDC2 | -0.926094291 | 6.08E-09 | down |
