# Supplementary material for: Investigation into the potential mechanism and molecular targets of Fufang Xueshuantong capsule for the treatment of ischemic stroke based on network pharmacology and molecular docking
Source: Front Pharmacol. 2022 Sep 15;13:949644. doi: 10.3389/fphar.2022.949644 (PMC9524248; doi:10.3389/fphar.2022.949644)
Supplement: Supplementary file 3 [file Table5.DOCX]

**SUPPLEMENTARY TABLE 5** The enrichment pathways corresponding to intersection genes.

| ID | Description | Gene ID | Count |
| --- | --- | --- | --- |
| hsa04913 | Ovarian steroidogenesis | PTGS2/CYP1B1/AKR1C3 | 3 |
| hsa05167 | Kaposi sarcoma-associated herpesvirus infection | PTGS2/STAT3/STAT1/HIF1A | 4 |
| hsa05235 | PD-L1 expression and PD-1 checkpoint pathway in cancer | STAT3/STAT1/HIF1A | 3 |
| hsa04659 | Th17 cell differentiation | STAT3/STAT1/HIF1A | 3 |
| hsa04066 | HIF-1 signaling pathway | STAT3/HIF1A/HK2 | 3 |
| hsa05145 | Toxoplasmosis | CD40LG/STAT3/STAT1 | 3 |
| hsa05206 | MicroRNAs in cancer | PTGS2/MMP9/STAT3/CYP1B1 | 4 |
| hsa00790 | Folate biosynthesis | AKR1B1/AKR1C3 | 2 |
| hsa00052 | Galactose metabolism | AKR1B1/HK2 | 2 |
| hsa00051 | Fructose and mannose metabolism | AKR1B1/HK2 | 2 |
| hsa05161 | Hepatitis B | MMP9/STAT3/STAT1 | 3 |
| hsa05205 | Proteoglycans in cancer | MMP9/STAT3/HIF1A | 3 |
| hsa05417 | Lipid and atherosclerosis | MMP9/CD40LG/STAT3 | 3 |
| hsa05208 | Chemical carcinogenesis - reactive oxygen species | CYP1B1/AKR1C3/HIF1A | 3 |
| hsa00140 | Steroid hormone biosynthesis | CYP1B1/AKR1C3 | 2 |
| hsa00590 | Arachidonic acid metabolism | PTGS2/AKR1C3 | 2 |
| hsa05321 | Inflammatory bowel disease | STAT3/STAT1 | 2 |
| hsa05204 | Chemical carcinogenesis - DNA adducts | PTGS2/CYP1B1 | 2 |
| hsa04917 | Prolactin signaling pathway | STAT3/STAT1 | 2 |
| hsa05230 | Central carbon metabolism in cancer | HIF1A/HK2 | 2 |
| hsa05212 | Pancreatic cancer | STAT3/STAT1 | 2 |
| hsa05140 | Leishmaniasis | PTGS2/STAT1 | 2 |
| hsa04657 | IL-17 signaling pathway | PTGS2/MMP9 | 2 |
| hsa04933 | AGE-RAGE signaling pathway in diabetic complications | STAT3/STAT1 | 2 |
| hsa04064 | NF-kappa B signaling pathway | PTGS2/CD40LG | 2 |
| hsa04625 | C-type lectin receptor signaling pathway | PTGS2/STAT1 | 2 |
| hsa04668 | TNF signaling pathway | PTGS2/MMP9 | 2 |
| hsa04935 | Growth hormone synthesis, secretion and action | STAT3/STAT1 | 2 |
| hsa04919 | Thyroid hormone signaling pathway | STAT1/HIF1A | 2 |
| hsa05162 | Measles | STAT3/STAT1 | 2 |
| hsa05160 | Hepatitis C | STAT3/STAT1 | 2 |
| hsa04217 | Necroptosis | STAT3/STAT1 | 2 |
| hsa04630 | JAK-STAT signaling pathway | STAT3/STAT1 | 2 |
