# Supplementary material for: Investigation into the potential mechanism and molecular targets of Fufang Xueshuantong capsule for the treatment of ischemic stroke based on network pharmacology and molecular docking
Source: Front Pharmacol. 2022 Sep 15;13:949644. doi: 10.3389/fphar.2022.949644 (PMC9524248; doi:10.3389/fphar.2022.949644)
Supplement: Supplementary file 4 [file Table3.DOCX]

**SUPPLEMENTARY TABLE 3** The 10 intersection genes sorted by logFC.

| Intersection gene | LogFC | *P* value |
| --- | --- | --- |
| PTGS2 | 0.604003932 | 3.164341742E-04 |
| AKR1B1 | -0.527407843 | 2.650366302E-06 |
| MMP9 | 1.350327617 | 2.363871339E-07 |
| CD40LG | -0.525838473 | 5.199633721E-06 |
| STAT3 | 0.518706000 | 9.802569049E-08 |
| STAT1 | 0.527018363 | 1.832962728E-04 |
| CYP1B1 | 0.777456051 | 3.466944015E-05 |
| AKR1C3 | -0.540589276 | 1.702679491E-03 |
| HIF1A | 0.522577291 | 2.901900525E-04 |
| HK2 | 0.710747842 | 6.247355524E-05 |
