# Supplementary material for: Investigation into the potential mechanism and molecular targets of Fufang Xueshuantong capsule for the treatment of ischemic stroke based on network pharmacology and molecular docking
Source: Front Pharmacol. 2022 Sep 15;13:949644. doi: 10.3389/fphar.2022.949644 (PMC9524248; doi:10.3389/fphar.2022.949644)
Supplement: Supplementary file 5 [file Table4.DOCX]

**SUPPLEMENTARY TABLE 4** Topological analysis results by degree-the first 33 proteins.

| Name | Description | Degree | Betweenness |
| --- | --- | --- | --- |
| STAT3 | signal transducer and activator of transcription 3 | 271 | 127.3203387 |
| STAT1 | signal transducer and activator of transcription 1 | 192 | 85.77427295 |
| NTRK1 | neurotrophic receptor tyrosine kinase 1 | 174 | 274.9765863 |
| HIF1A | hypoxia inducible factor 1 alpha subunit | 172 | 138.3467529 |
| TP53 | tumor protein p53 | 141 | 139.5057255 |
| HSP90AA1 | heat shock protein 90 alpha family class A member 1 | 141 | 278.7212128 |
| EGFR | epidermal growth factor receptor | 131 | 151.3544931 |
| ESR1 | estrogen receptor 1 | 131 | 151.3924873 |
| EP300 | E1A binding protein p300 | 122 | 70.93726756 |
| HSP90AB1 | heat shock protein 90 alpha family class B member 1 | 103 | 153.2545266 |
| SRC | SRC proto-oncogene, non-receptor tyrosine kinase | 102 | 90.46885644 |
| MDM2 | MDM2 proto-oncogene | 99 | 82.80737595 |
| VCP | valosin containing protein | 98 | 126.7793543 |
| HDAC1 | histone deacetylase 1 | 95 | 75.56784176 |
| AR | androgen receptor | 92 | 104.8519976 |
| XPO1 | exportin 1 | 90 | 60.08037062 |
| BRCA1 | BRCA1, DNA repair associated | 90 | 124.2598385 |
| RELA | RELA proto-oncogene, NF-kB subunit | 90 | 71.20384954 |
| CTNNB1 | catenin beta 1 | 89 | 167.6348801 |
| HSPA8 | heat shock protein family A (Hsp70) member 8 | 89 | 149.5518209 |
| TRAF6 | TNF receptor associated factor 6 | 88 | 81.75740982 |
| VHL | von Hippel-Lindau tumor suppressor | 84 | 103.5767611 |
| MYC | v-myc avian myelocytomatosis viral oncogene homolog | 82 | 76.63501559 |
| HSPA4 | heat shock protein family A (Hsp70) member 4 | 80 | 144.4192366 |
| TRIM28 | tripartite motif containing 28 | 69 | 102.7042193 |
| PML | promyelocytic leukemia | 65 | 76.11930765 |
| PARP1 | poly(ADP-ribose) polymerase 1 | 63 | 86.93200173 |
| HDAC3 | histone deacetylase 3 | 61 | 47.82700019 |
| ENO1 | enolase 1 | 57 | 60.32685587 |
| NR3C1 | nuclear receptor subfamily 3 group C member 1 | 57 | 66.48160593 |
| PPP1CA | protein phosphatase 1 catalytic subunit alpha | 52 | 53.32267829 |
| MAPK8 | mitogen-activated protein kinase 8 | 49 | 48.21919863 |
| RUVBL2 | RuvB like AAA ATPase 2 | 47 | 55.75931875 |
