# Supplementary material for: Investigation into the potential mechanism and molecular targets of Fufang Xueshuantong capsule for the treatment of ischemic stroke based on network pharmacology and molecular docking
Source: Front Pharmacol. 2022 Sep 15;13:949644. doi: 10.3389/fphar.2022.949644 (PMC9524248; doi:10.3389/fphar.2022.949644)
Supplement: Supplementary file 6 [file Table6.DOCX]

**SUPPLEMENTARY TABLE 6** Virtual docking results of core target-chemical components of FFXST in treatment of ischemic stroke

| Core target | Chemical composition | Binding energy/kcal•mol^-1^ |
| --- | --- | --- |
| STAT1 | kaempferol | -6.9 |
| STAT1 | quercetin | -7.2 |
| STAT3 | crytotanshinone | -8.7 |
| HIF1A | quercetin | -7.5 |
